# A State Sanatorium for Consumptives

**Published:** 1900-12

**Authors:** 


### TEXAS MEDICAL JOURNAL.

AUSTIN, TEXAS.

A MONTHLY JOURNAL OF MEDICINE AND SURGERY.

EDITED AND PUBLISHED BY

F. E. DANIEL, M. D., AND S. E. HUDSON, M. D.

Published Monthly at Austin, Texas, by Drs. Daniel and Hudson. Subscription price $1.00 a year in advance.

Eastern Representative: John Guy Monihan, St. Paul Building, 220 Broadway, New York City.

Official organ of the West Texas Medical Association, the Houston District Medical Association, the Austin District Medical Society, the Brazos Valley Medical Association, the Galveston County Medical Society, and several others.

A Merry Christmas, Doctor! The "Red Back" kisses its hand to all its readers, and sends them greeting, wishing all a merry Christmas and lots of egg-nogg. May great chunks of "prosperity" come their way—come to each and every one of the hard-worked and tired doctors, and stop long enough for them to get a slice. The doctors ought to feel good this year,—cotton 10 cents, and collections good. We hope that they may follow Mr. McKinley's policy of "benevolent assimilation," and assimilate great quantities of greenbacks.

### A STATE SANATORIUM FOR CONSUMPTIVES.

The committee appointed by the Texas State Medical Association to endeavor to secure the necessary legislation for a State sanatorium for indigent consumptives consists of Dr. Frank Paschall, San Antonio, chairman; Dr. D. R. Fly, Amarillo; Dr. M. M. Smith, Austin; Dr. F. M. Pitts, Hubbard City, and Dr. Taylor Hudson, Belton. Dr. F. E. Daniel, Austin, Texas, has been added to the committee by request. Drs. Paschall, Smith and Daniel had a very satisfactory interview with Governor Sayers recently. The Governor expressed a deep interest in the subject, and requested the committee to submit their views in writing, promising to give it careful attention.

The danger of contracting consumption is not appreciated by the people. Its very insidiousness makes it the more dangerous and the more difficult to deal with. Were it acutely contagious, as are smallpox, scarlet fever, diphtheria, etc.; as infectious as yellow fever, the machinery of the State—that is, of the quarantine department,—would be put in operation to prevent its introduction into the State and its spread among the people; but in the present state of public sentiment and ignorance of the danger, it is out of the question to talk of quarantining against it, or of putting a consumptive in a quarantine camp, as is done with smallpox, etc., and with those who have been exposed to it, or to any other infection or contagion except consumption. Attracted by the mild winter climate of Southern Texas, thousands of consumptives flock to the State. Thousands of the very poor, afflicted with consumption, drift in, and being without means or friends, they are found often in San Antonio and other Texas cities destitute and suffering; frequently they are taken from the streets and sent to the city hospital. Humanity demands this, yet it is, in itself, a great danger,—danger to the inmates of the hospital and danger to the community at large. What else can be done? It is not possible to stop them at the State line and refuse them admittance, or quarantine them, as said before; they cannot be sent back to the place from whence them came. It is not possible to "vaccinate" or inoculate against the infection. What recourse, then, is left the State of Texas for the protection of its people, and for the amelioration of the suffering of these poor creatures, and to prevent the dissemination of the seeds of the disease, except to "corral" them, to use a Texas expression? To care for them in a sanatorium or sanatoria at some point in Texas, in a mild and dry climate, remote from the business centers and dense population seems to offer the only solution to the problem. The whole of that grand elevated plateau between the Pecos and the Rio Grande (and East of it as far as Kendall, Edwards and other counties), furnishes the ideal climate for consumptives. It is well known that so dry is the atmosphere in that section that a beef killed, even in summer, and hung up in the open air will not decompose; the atmosphere it fatal to most pathogenic germs, and putrefaction does not take place. Some day this region, when it becomes known, as it will be in time, will constitute one grand sanitarium for the world.

If the State can be induced to establish a hospital or hospitals for consumptive poor—as has been suggested to the Governor, and will be urged before the Legislature by the committee above mentioned, in a short while such institutions could, under proper management, be made self-sustaining by the industry of the inmates, who would be greatly benefited by an active out-door life—and light labor—as on a ranch, market or dairy garden, for such of them as are able to work.

We can not go fully into a discussion of the subject here; but hope in our next issue to give a brief of the arguments that will be submitted to the Governor—at his request.

